# Supplementary material for: End-Triassic mass extinction started by intrusive CAMP activity
Source: Nat Commun. 2017 May 31;8:15596. doi: 10.1038/ncomms15596 (PMC5460029; doi:10.1038/ncomms15596)
Supplement: Supplementary Information — Supplementary Figures and Supplementary References [file ncomms15596-s1.pdf]

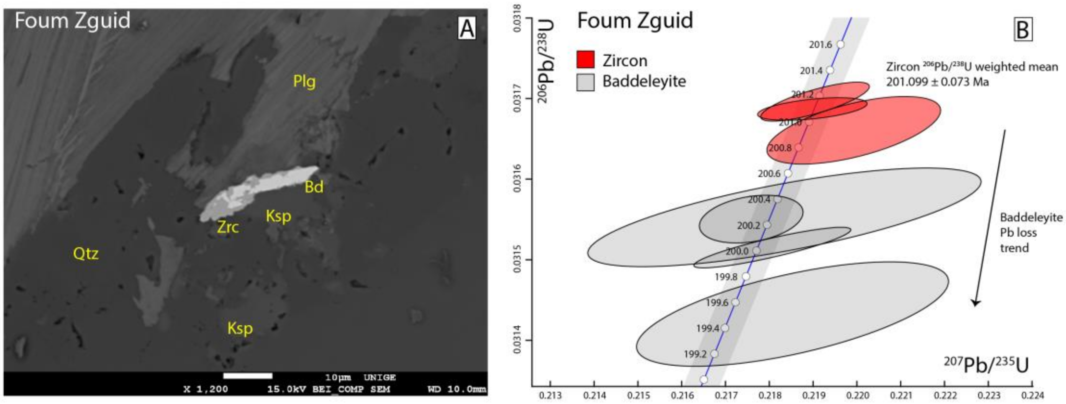

Supplementary Figure 1

A) Backscattered electron image of a highly evolved melt pocked in the Foum Zguid dyke that has crystalized baddeleyite (Bd) and zircon (Zrc) surrounded by plagioclase (Plg), k-feldspar (Ksp) and quartz (Qtz). Note that the Foum Zguid dyke is gabbroic. B) Concordia plot for the Foum Zguid dyke showing chemically abraded zircon ages relative to baddeleyite, uncertainty ellipses are shown at 2σ. The thin section image suggests that baddeleyite and zircon co-crystalized, or that zircon postdates the baddeleyite. The apparently younger baddeleyite ages are interpreted to reflect Pb loss and are not included in the final age. This figure is a modified version of Fig 11 from [1].

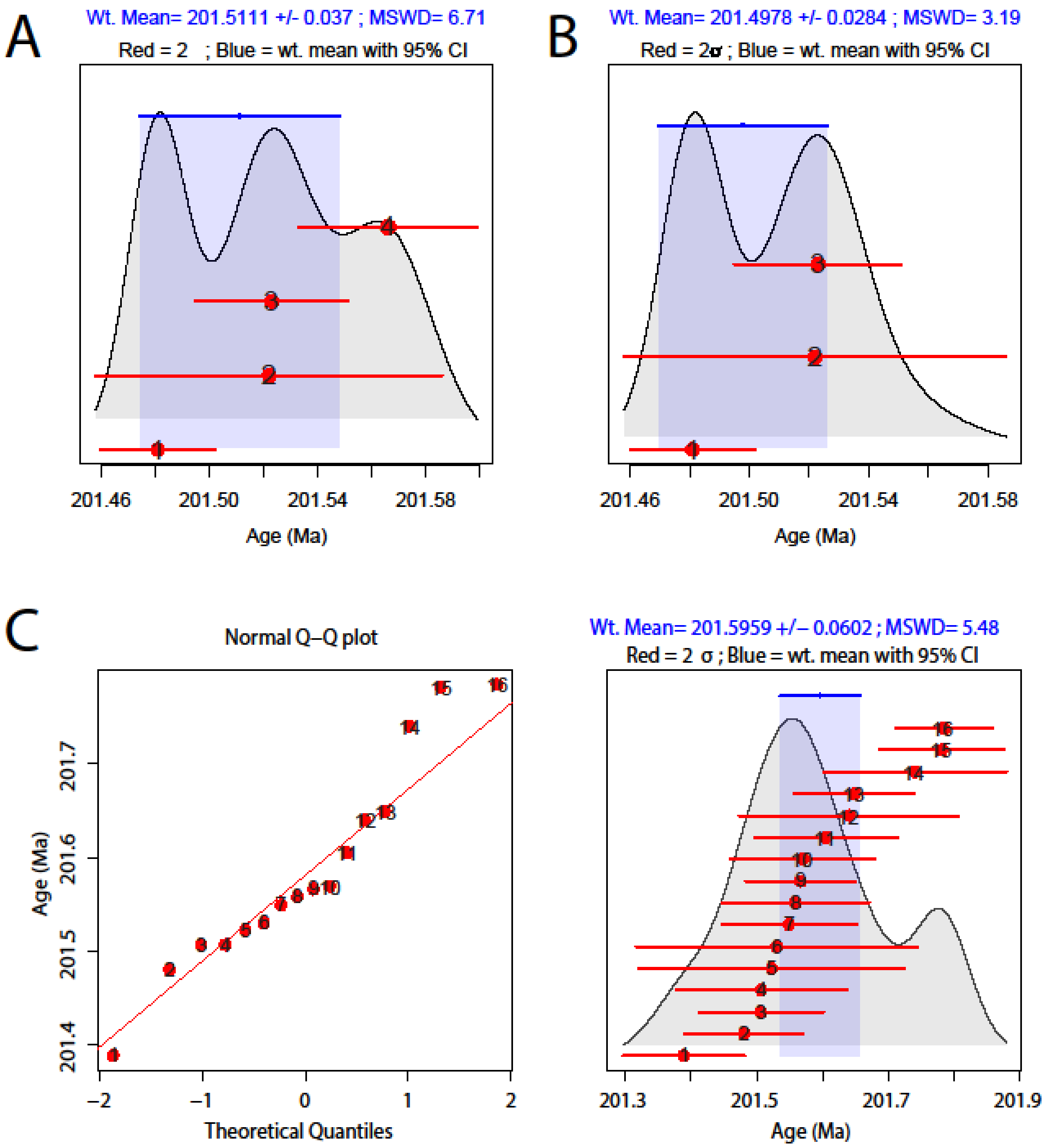

Supplementary Figure 2

Weighted mean plots with associated probability density functions for the North Mountain basalt. A) Weighted mean age for the all high precision U-Pb NMB ages arranged in date order, 1 is from ref. [3], recalculated using the more recent spike composition [7], 2 is the Westport drill hole sample [2], 3 is the NMB age from this study, 4 the preferred NMB age from [2]. Note that ages 1,3 and 4 are from zircons in pegmatite lenses in the same quarry in the NMB. B) The preferred NMB age

calculated in this study, a combination of the age from ref. [3] the Westport drill hole age from ref [2] and the age from this study. C) All of the analysis from the age preferred by ref. [2] showing the analyses potentially affected by micro cores – grains 14, 15 and 16, the Q-Q plot is also shown to further support the interpretation that these analyses are outliers.

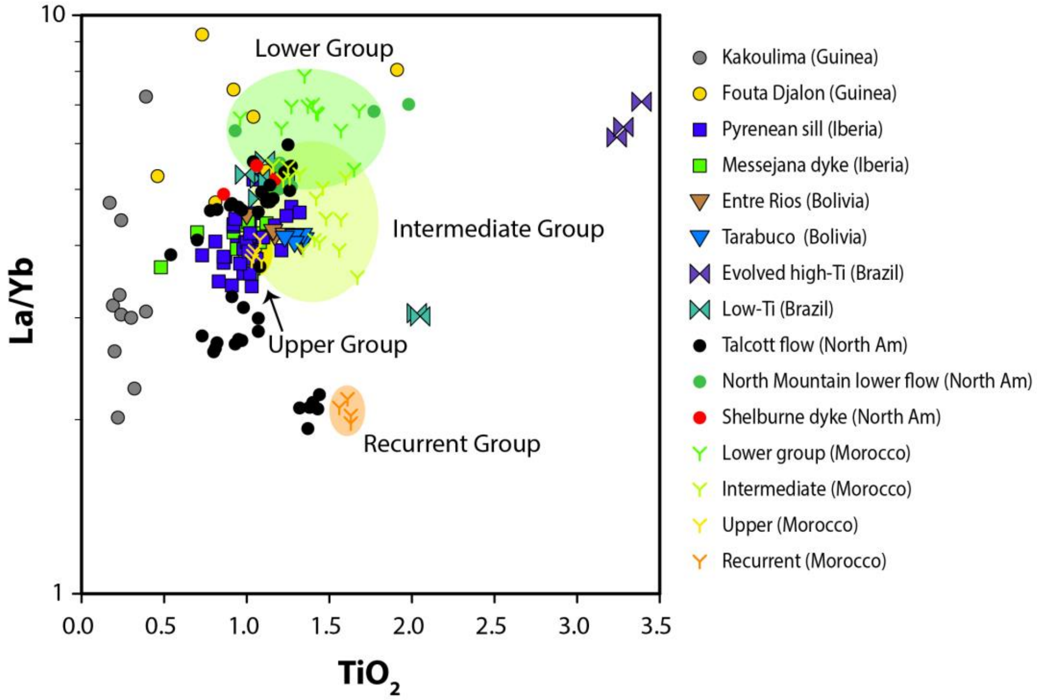

Supplementary Figure 3

Geochemistry of the CAMP intrusives dated in this study (apart from Hodh), compared with the geochemical groupings from the Moroccan lava flows. All of the geochemical analyses have been previously published [5-18]. Note the logarithmic Y-axis.

Supplementary references

1. Schaltegger, U. & Davies, J.H.F.L. in *Petrochronology* (ed. Kohn, M & Engi, M) (Reviews in Mineralogy and Geochemistry, vol. **83,** 2017)
2. Schoene, B., Guex, J., Bartolini, A., Schaltegger, U. & Blackburn, T. J. Correlating the end-Triassic mass extinction and flood basalt volcanism at the 100 ka level. *Geology* **38,** 387–390 (2010).

3. Blackburn, T. J. *et al.* Zircon U-Pb Geochronology Links the End-Triassic Extinction with the Central Atlantic Magmatic Province. *Science* **340,** 941–945 (2013).
4. Wotzlaw, J.-F. *et al.* Towards accurate numerical calibration of the Late Triassic: High-precision U-Pb geochronology constraints on the duration of the Rhaetian. *Geology* **42,** 571–574 (2014).
5. Deenen, M. H. L. *et al.* A new chronology for the end-Triassic mass extinction. *EPSL* **291,** 113–125 (2010).
6. Bertrand, H. in *Magmatism in Extensional Structural Settings* 147–188 (Springer Berlin Heidelberg, 1991). doi:10.1007/978-3-642-73966-8_7
7. Marzoli, A. *et al.* Synchrony of the Central Atlantic magmatic province and the Triassic-Jurassic boundary climatic and biotic crisis. *Geology* **32,** 973–976 (2004).
8. Youbi, N. *et al.* in *The Central Atlantic Magmatic Province: Insights From Fragments of Pangea* **136,** 179–207 (American Geophysical Union, 2003).
9. Callegaro, S. *et al.* Upper and lower crust recycling in the source of CAMP basaltic dykes from southeastern North America. *EPSL* **376,** 186–199 (2013).
10. Merle, R. *et al.* Sr, Nd, Pb and Os Isotope Systematics of CAMP Tholeiites from Eastern North America (ENA): Evidence of a Subduction-enriched Mantle Source. *JPet* **55,** 133–180 (2014).
12. Deckart, K., Bertrand, H. & Liégeois, J.-P. Geochemistry and Sr, Nd, Pb isotopic composition of the Central Atlantic Magmatic Province (CAMP) in Guyana and Guinea. *Lithos* **82,** 289–314 (2005).
13. Bertrand, H., Dostal, J. & Dupuy, C. Geochemistry of early Mesozoic tholeiites from Morocco. *EPSL* **58,** 225–239 (1982).
14. De Min, A. *et al.* The Central Atlantic Magmatic Province (CAMP) in Brazil: Petrology, Geochemistry, 40Ar/39Ar Ages, Paleomagnetism and Geodynamic Implications. *Large Igneous Provinces; Continental, Oceanic, and Planetary Flood Volcanism* **136,** 91–128 (2003).
15. Cebriá, J. M., López-Ruiz, J., Doblas, M., Martins, L. T. & Munha, J. Geochemistry of the Early Jurassic Messejana–Plasencia dyke (Portugal–Spain); Implications on the Origin of the Central Atlantic Magmatic Province. *JPet* **44,** 547–568 (2003).
16. Bertrand, H., Fornari, M., Marzoli, A., García-Duarte, R. & Sempere, T. The Central Atlantic Magmatic Province extends into Bolivia. *Lithos* **188,** 33–43 (2014).
17. Merle, R. *et al.* 40Ar/39Ar ages and Sr–Nd–Pb–Os geochemistry of CAMP tholeiites from Western Maranhão basin (NE Brazil). *Lithos* **122,** 137–151 (2011).
18. Callegaro, S. *et al.* Enriched mantle source for the Central Atlantic magmatic province: Newsupporting evidence from southwestern Europe. *Lithos* **188,** 15–32 (2014).
